# Eosinophil: A Nonnegligible Predictor in COVID-19 Re-Positive Patients

**DOI:** 10.3389/fimmu.2021.690653

**Published:** 2021-07-29

**Authors:** Xiaolu Li, Deqing Yin, Yanyan Yang, Chunhua Bi, Zhibin Wang, Guangren Ma, Xiuxiu Fu, Shengxiang Ji, Fachun Jiang, Tao Yu

**Affiliations:** ^1^Department of Cardiac Ultrasound, The Affiliated Hospital of Qingdao University, Qingdao, China; ^2^Department of Microbiology, Linyi Center for Disease Control and Prevention, Linyi, China; ^3^Department of Immunology, School of Basic Medicine, Qingdao University, Qingdao, China; ^4^Department of Infectious Disease, The Affiliated Hospital of Qingdao University, Qingdao, China; ^5^Department of Medical Education, Qingdao Chest Hospital, Qingdao, China; ^6^Department of Infectious Disease, Qingdao Centre for Disease Control and Prevention, Qingdao Institute of Prevention Medicine, Qingdao, China; ^7^Institute for Translational Medicine, The Affiliated Hospital of Qingdao University, Qingdao, China

**Keywords:** COVID-19, SARS-CoV-2, re-positive, eosinophils, clinical index

## Abstract

Although vaccine resources are being distributed worldwide, insufficient vaccine production remains a major obstacle to herd immunity. In such an environment, the cases of re-positive occurred frequently, and there is a big controversy regarding the cause of re-positive episodes and the infectivity of re-positive cases. In this case-control study, we tracked 39 patients diagnosed with COVID-19 from the Jiaodong Peninsula area of China, of which 7 patients tested re-positive. We compared the sex distribution, age, comorbidities, and clinical laboratory results between normal patients and re-positive patients, and analysed the correlation between the significantly different indicators and the re-positive. Re-positive patients displayed a lower level of serum creatinine (63.38 ± 4.94 U/L *vs*. 86.82 ± 16.98 U/L; P =0.014) and lower albumin (34.70 ± 5.46 g/L *vs*. 41.24 ± 5.44 g/L, P =0.039) at the time of initial diagnosis. In addition, two positive phases and the middle negative phase in re-positive patients with significantly different eosinophil counts (0.005 ± 0.005 × 10^9^/L; 0.103 ± 0.033 × 10^9^/L; 0.007 ± 0.115 × 10^9^/L; Normal range: 0.02-0.52 × 10^9^/L). The level of eosinophils in peripheral blood can be used as a marker to predict re-positive in patients who once had COVID-19.

## Introduction

On 11 February 2020, the World Health Organization (WHO) officially named the emerging infectious disease that broke out in Wuhan, China in December 2019 as coronavirus disease-19 (COVID-19). Subsequently, the aetiological agent was identified by the Coronavirus Study Group of the International Committee on Taxonomy of Viruses, and the virus causing this severe respiratory disease was named as severe acute respiratory syndrome coronavirus 2 (SARS-COV-2) (1). During the next few months, COVID-19 rapidly spread worldwide to become the highest threat caused by a pandemic. According to the latest statistics from the Johns Hopkins University, by August 16, 2020, the total number of COVID-19 cases globally exceeds 21.48 million, with over 771,000 deaths (2). The data is constantly updated with a rising infection and mortality rate reported. It is estimated that the global pandemic would last until June 2021, with intermittent lockdowns considered the ‘new normal’ (3). Additionally, it is estimated that 250 million people would be infected and 1.75 million deaths would occur worldwide by June 2021. Alarmingly, variants of the SARS-CoV-2 strain have been found in many countries around the world. However, there are still many controversies regarding the relationship between infectivity, pathogenicity, and the association between variation of the novel strain and the recurrence of the disease in patients (re-positive state). If the patient’s re-positive state cannot be effectively controlled, the prevention and control of the global pandemic and the allocation of medical resources will face greater stress for an unpredictable duration. In addition, clinicians and scholars are now concerned whether COVID-19 will become a refractory chronic disease due to the patient’s repeated re-positive episodes.

Since the outbreak of the disease, many research articles on clinical cases have focused on epidemiology, clinical features, laboratory examination, and imaging features, to promote the development of diagnosis and treatment of clinical patients. The criteria for Chinese COVID-19 patients to be cured and discharged include recovered of body temperature, improvement of respiratory symptoms, improvement of lesions by radiological examination, and negative viral nucleic acid test of respiratory specimens for two consecutive tests (4). However, it is worth noting that since February 2020, re-positive cases have been reported in succession (5–9). Especially at a time when vaccines are being distributed and administered quickly, there are cases of patients reactivating. Patients are defined re-positive if they meet the discharge criteria and show positive viral nucleic acid test results from pharyngeal swabs or rectal swab samples, during a continuous review after discharge. The appearance of these cases has attracted a lot of attention, as it is presently unknown whether returning re-positive patients can transmit the virus. In addition, there is no clinical and experimental data to determine whether the cause of a patient’s re-positive state is a false negative test result or virus re-positive or re-infection.

In this study, a total of 39 COVID-19 patients in Qingdao were retrospectively analysed, among which 7 patients developed a re-positive state. We studied the differences in epidemiological characteristics and clinical laboratory parameters, including blood count and serum biochemical examination, between re-positive patients and recovered patients, and at different phases of re-positive patients. We founded that male over the age of 45 year old appeared to account for a greater proportion of the re-positive patients from the Jiaodong Peninsula area. Both creatinine and eosinophils counts can be used as a marker to predict re-positive in patients who once had COVID-19. We hope to comprehensively summarize the significant characteristics of re-positive patients, to provide a reliable theoretical basis for clinical prediction, treatment, and effective control of re-positive episodes, and to provide a breakthrough for further in-depth studies of the causes of re-positive episodes and the mechanism of viral immunity.

## Materials and Methods

### Study Design and Patients

This case-control study included 39 patients diagnosed with COVID-19 admitted to The Affiliated Hospital of Qingdao University and Qingdao Chest Hospital from January 18 to November 7, 2020. The diagnosis, clinical classification (mild, medium, severe, and critical), and discharge criteria for patients with COVID-19 pneumonia are based on the COVID-19 Prevention and Control Plan (7th edition) published by the National Health Commission (3). Patients who met the required standards and were allowed to leave the hospital, were quarantined for 14 days as required, and were tested for SARS-COV-2 nucleic acid in a timely manner. From these patients, re-positive patients were admitted to the hospital again, and a detailed routine blood and serum biochemical examination was conducted, and their close contacts were isolated for observation.

### Data Collection

The clinical records of 39 patients with COVID-19, including 7 re-positive patients, were reviewed. The clinical data obtained during the patient’s hospitalization were collected from electronic medical records by two trained researchers. We collected clinical data including age, sex, symptoms, comorbidities, laboratory test results, details of treatment, and clinical outcomes. All clinical data were independently examined and entered into the database by two investigators, and the final review and evaluation of the input data was performed by a third investigator. The patient’s clinical results were followed until November 7, and patients had only one re-positive.

### RT-PCR Test

Positive results of reverse transcription PCR (RT-PCR) assays were used to confirm SARS-CoV-2 infection and define re-positive patients. Deep nasal swab or throat swab samples were collected, from which SARS-CoV-2 RNA was extracted. A SARS-COV-2 nucleic acid detection kit was used, and RT-PCR detection was performed according to manufacturer’s instructions. The RT-PCR amplified the SARS-CoV-2 genome’s open reading frame lab (ORF1ab) and nucleocapsid protein (N) regions, and the cycle threshold value was determined according to the manufacturer’s instructions. The details are as follows: ORF1ab: forward, 5’-CCCTGTGGGTTTTACACTTAA-3’, reverse,5’-ACGATTGTGCATCAGCTGA-3’ and Fluorescence, 5’-FAM-CCGTCTGCGGTATGTGGAAAGGTTATGG-BHQ1-3’; N: forward, 5’-GGGGAACTTCTCCTGCTAGAAT-3’, reverse, 5’-CAGACATTTTGCTCTCAAGCTG-3’ and Fluorescence, 5’-FAM-TTGCTGCTGCTTGACAGATT-TAMRA-3’. The results of RT-PCR with no Ct value or a Ct value is 40 were reported as negative, while those with a Ct value less than 37 were reported as positive. When the Ct value is between 37 and 40, the result is considered suspicious positive. In addition, any suspected positive result was tested again. The sample is judged to be positive if the repeat results Ct value is less than 40, and amplification curve has obvious peaks, otherwise it is negative.

### Statistical Analysis

Statistical analysis was performed using SPSS22.0 (IBM, Chicago). Descriptive statistics were generated to characterize the study population. Continuous variables were described as the mean with standard deviation or median with interquartile range. Univariable analysis between different groups was done using Student’s t-test and Kolmogorov-Smirnov test. Paired t-test was used to analyse the difference of clinical indicators of re-positive patients in the case of RT-PCR negative test and positive test, Next, factors with statistically significance (P<0.05) were further analysed by ANOVA for single factor repeated measurements followed by Bonferroni correction at different stages, and verified by the logistic cubic curve regression model. Results from the correlation are reported as p value. All reported p values are two-sided.

## Results

### Demographic and Epidemiological Characteristics

A total of 39 patients diagnosed with COVID-19 were discharged from the hospital between 29^th^ January and 26^th^ May, including 1 death, 5 severe cases, 21 non-severe cases, and 1 asymptomatic case. The discharged patients were followed up with for check-up for at least 14 days. As of June 9, 7 re-positive patients were found, comprising 2 female (28.6%) and 5 male (71.4%) patients. The mean age was 53.14 ± 21.65 years; 2 patients (28.6%) were younger than 45 years, whereas 5 patients (71.4%) were older than 45 years. In recovered patients who did not turn re-positive, 12 patients (37.5%) were male and 20 patients (62.5%) were female. The mean age was 46.69 ± 18.36 years, where 15 patients (46.9%) were younger than 45 years and 17 patients (53.1%) were older than 45 years. There were no significant differences in age (P =0.484) or sex distribution (P =0.205) between the re-positive and recovered groups. Of these re-positive patients, 6 (85.7%) had at least one comorbid condition listed in which hypertension, hyperlipidaemia, diabetes, and chronic bronchitis. History of sick contact was present in all patients. During the initial diagnosis of COVID-19 in the re-positive groups, there were 2 severe cases and 5 non-severe cases. The date of onset of re-positive episodes ranged from January 27 to March 6, 2020. The mean duration of the first RT-PCR positive period duration was 13.67 (10-18) days, and the mean duration of the subsequent RT-PCR negative period was 22.67 (6-47) days. The mean duration of RT-PCR re-positive prior was 10.40 (2-18) days. In one patient, the symptoms during the re-positive period were more severe than during the initial infection, but the clinical examination showed that it might be associated with bacterial infection. The symptoms of the other patients during the re-positive period were milder than in their initial infection, or there were no visible clinical symptoms during the re-positive period, except for the positive RT-PCR test results (**Table 1**).

**Table 1:** Baseline characteristics of re-positive patients with COVID-19.

Characteristic	Re-positive group (n = 7)	recovered group (n = 32)	Statistic	p-Value
Male [(n,%)]	5 (71.43)	12 (37.50)	X^2 ^= 2.69	0.205
Age [year($\bar{\text{X}}$ ± s)]	53.14 ± 21.65	46.69 ± 18.36	t = 0.733	0.484
Comorbidities [(n,%)]				
Cardiovascular disease	4 (57.14)	7 (21.88)	X^2 ^= 3.53	0.083
Diabetes	2 (28.57)	0	NA	NA
Chronic bronchitis	1 (14.29)	0	NA	NA

Data are mean ± SD, n/N (%), where NA is the total number of patients with available data. Cardiovascular disease: hypertension, hyperlipidaemia, and coronary atherosclerotic cardiopathy.

### Differences Between Re-Positive and Recovered Patients

**Figure 1:**
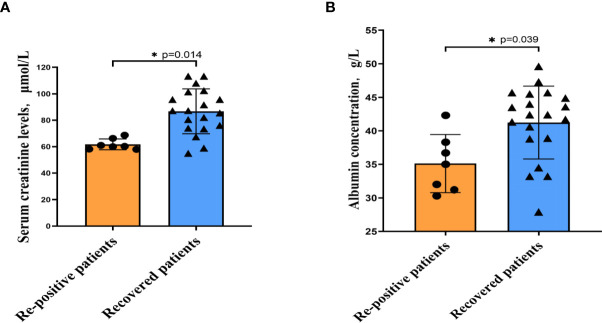
The indicators of significant difference between patients with and without re-positive. Re-positive patients had lower serum creatinine concentrations **(A)** and albumin concentrations **(B)** at the time of initial admission than did recovered patients. Yellow represents the re-positive patients, and blue represents the recovered patients. All quantification graphs represent mean ± SD.

To study the differences between the re-positive patients and recovered patients, we compared and analysed the routine blood and serum biochemical indexes at admission, and found that the creatinine levels of re-positive patients were significantly lower than that of recovered patients (63.38 ± 4.94 U/L *vs*. 86.82 ± 16.98 U/L; P =0.014). Creatinine is a marker of kidney injury, and viral infection can lead to increased creatinine levels. Lower creatinine levels were observed in re-positive patients at the time of initial diagnosis, which cannot be explained. However, these results suggest that creatinine levels at the time of initial diagnosis may be a potential indicator of developing re-positive episodes in the future. Another indicator of a significant difference was a lower level of serum albumin concentration in patients with re-positive patients (34.70 ± 5.46 g/L *vs*. 41.24 ± 5.44 g/L, P =0.039) (**Figure 1**). In severe patients with COVID-19, a variety of inflammatory indicators will be increased. Some indicators have been reported in published articles to assess the severity and predict the prognosis of COVID-19 (10–13). In this study, we compared inflammatory indicators, including C-reactive protein (CRP), white blood cell count, neutrophil count, lymphocyte count, and Neutrophil-to-lymphocytes ration (NLR), and the results show that there is no significant difference in these indicators between the patients who are re-positive and those who are not (**Table 2**).

**Table 2:** Comparison of laboratory indicators between re-positive and recovered group.

Variable category	Re-positive group	Recovered group	Statistic	p-Value
Organ functional indicators				
Total protein [g/L($\bar{\text{X}}$ ± s)]	60.70 ± 5.37	67.18 ± 7.30	t=1.663	0.112
Albumin* [g/L($\bar{\text{X}}$± s)]	34.70 ± 5.46	41.24 ± 5.44	t=2.190	0.039
Total bilirubin [μmol/L($\bar{\text{X}}$± s)]	10.73 ± 3.80	14.22 ± 5.47	t=1.205	0.242
Aspertate aminotransferase [U/L($\bar{\text{X}}$ ± s)]	28.68 ± 12.50	28.74 ± 21.28	t=0.006	0.995
Alanine aminotransferase [U/L($\bar{\text{X}}$ ± s)]	23.63 ± 5.10	31.89 ± 5.05	t=0.289	0.775
Serum urea [mmol/L($\bar{\text{X}}$ ± s)]	4.85 ± 1.68	3.98 ± 0.95	t=-1.465	0.158
Serum creatinine* [μmol/L($\bar{\text{X}}$± s)]	63.38 ± 4.94	86.82 ± 16.97	t=2.693	0.014
Inflammation indicators				
CRP [mg/L(IQR)]	21.80 (2.94-53.57)	26.91 (8.32-36.00)	t=-0.373	0.717
White blood cell [×10^9^/L($\bar{\text{X}}$± s)]	6.43 ± 1.84	5.42 ± 1.83	t=1.313	0.221
Neutrophil [×10^9^/L($\bar{\text{X}}$± s)]	4.45 ± 1.68	3.37 ± 1.90	t=1.492	0.167
Lymphocyte [×10^9^/L($\bar{\text{X}}$± s)]	1.44 ± 0.76	1.41 ± 0.75	t=0.100	0.922
NLR (IQR)	4.28 (1.52-6.22)	3.34 (1.65-3.84)	t=0.626	0.548

Data are mean ± SD, n/N (%), where NA is the total number of patients with available data. CRP, C-reactive protein; NLR, neutrophil-to-lymphocytes ration. *P < 0.05

For all demographic data, clinical characteristics and laboratory findings in univariate analysis, we identified each variable that showed and/or reached statistical significance with p<0.05 between the patients who are re-positive and those who are not. For Pearson correlation analyses, variables initially entered the model included serum creatinine level and serum albumin concentration. Results showed that serum creatinine level was the only significant variable (p =0.014) and it can be independently predicted for re-positive of COVID-19.

### Characteristics of Clinical Indicators in Re-Positive Patients

**Figure 2:**
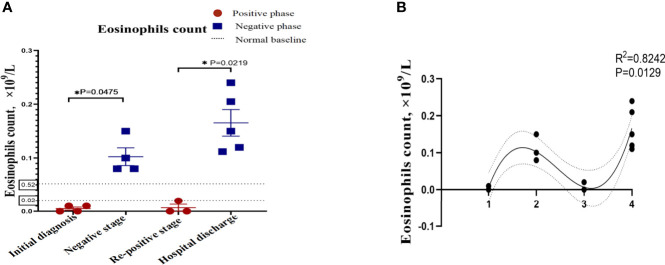
The indicators of significant difference between patients with and without re-positive. Re-positive patients had lower serum creatinine concentrations **(A)** and albumin concentrations **(B)** at the time of initial admission than did recovered patients. Yellow represents the re-positive patients, and blue represents the recovered patients. all quantification graphs represent mean ± SD.

**Figure 3:**
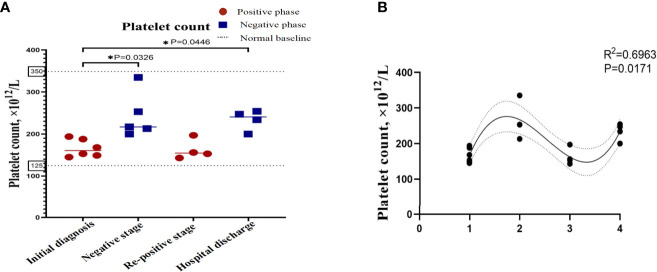
Platelet counts in re-positive patients of COVID-19 at different phases. The platelet count of the patients with re-positive was lower than that of the negative stage in the initial stage and the re-positive stage, and the difference was statistically significant. **(A)** Platelet count at different stages of the patients. The X-axis represents the stage of the patient's course, which is segmented based on RT-PCR results, and the Y-axis is the value of the index. The red dots represent the positive results of RT-PCR and the blue dots represent the negative results of RT-PCR. The values are the mean values. Data that is not shown is not available. **(B)** Correlation between platelet count and RT-PCR results. R2 represents the degree of fit between the data points and the curve. The number on X-axis represents four rounds of RT-PCR.

In order to describe the clinical laboratory characteristics of re-positive patients at different stages and identify the clinical indicators that can be associated with re-positive episodes, we analysed the blood routine and serum biochemistry of re-positive patients. The results showed that eosinophil counts decreased significantly to abnormal values during both the initial diagnosis and the re-positive episode (0.005 ± 0.005 × 10^9^/L; 0.103 ± 0.033 × 10^9^/L; 0.007 ± 0.115 × 10^9^/L; Normal range: 0.02-0.52 × 10^9^/L). We then analysed the difference in eosinophil counts in re-positive patients at different stages (initial diagnosis, recurrent negative stage, and recurrent positive stage), and found that a significant difference existed between the recurrent negative stage and the other two stages (P =0.002) (**Figure 2**). In addition, there was no abnormal platelet counts in the three stages (166.17 ± 20.84 × 10^12^/L: 267.00 ± 62.19 × 10^12^/L: 162.25 ± 23.82 × 10^12^/L), but we found that the platelet count of re-positive patients in the negative phase was significantly higher than that in the other two phases (P =0.004). These findings suggest that platelet counts and eosinophil counts appear to indicate the course of the COVID-19 disease in patients, especially in re-positive patients (**Figure 3**). Since the symptoms of the re-positive period were milder than that of the initial diagnosis period, we specifically compared the inflammatory indicators of the two periods and found no significant difference (**Table 3**).

**Table 3:** Comparison of inflammatory indicators between re-positive phase and initial diagnosis phase.

Variable category	Re-positive phase	Initial diagnosis phase	Statistic	p-Value
CRP [mg/L(IQR)]	NA	21.80 (2.94-53.57)		
white blood cell [×10^9^/L($\bar{\text{X}}$ ± s)]	8.63 ± 4.29	6.43 ± 1.84	t=1.188	0.269
neutrophil [×10^9^/L($\bar{\text{X}}$ ± s)]	6.37 ± 4.40	4.45 ± 1.68	t=0.840	0.454
Lymphocyte [×10^9^/L($\bar{\text{X}}$ ± s)]	1.10 ± 0.81	1.44 ± 0.76	t=-0.713	0.494
NLR (IQR)	14.21 (1.97-33.92)	4.28 (1.52-6.22)	t=1.416	0.190

Data are mean±SD, n/N (%), where NA is the total number of patients with available data. CRP, C-reactive protein; NLR, Neutrophil-to-lymphocytes rations.

We identified that eosinophil counts and platelet count showed reached statistical significance with p<0.05 among the different phases of re-positive patients. After one-way ANOVA analyses, results showed that eosinophil counts (**Figure 2B**) and platelet counts (**Figure 3B**) were significant variable which could indicate the progression of patient’s course of COVID-19.

## Discussion

The outbreak of COVID-19 has caused a global crisis, as the disease continues to spread with the number of cases increasing rapidly. Despite the emergence of candidate vaccines (14), it remains to be seen whether the vaccines can continue to effectively protect the population from infection and prevent re-positive episodes in the context of rapid viral mutations. In the absence of specific drugs and natural immunity, the re-positive episodes of patients discharged from hospitals have a negative impact on the treatment and control of the disease, and mental health of the patients. Therefore, it is particularly important to master the monitoring indices for the prediction of the rate of recurrence in order to successfully determine the causes of the re-positive episodes.

The patients tracked in this study were treated in accordance with the guidelines followed during their first confirmed hospitalization and were discharged after meeting the criteria. After discharge, all patients were strictly quarantined and observed, where re-positive patients were isolated from other infected patients. Therefore, we excluded the cases of patients re-infected with the virus, and selected six patients that were re-positive after treatment of their first viral infection. Presently, there is a lack of conclusive evidence on why patients have developed re-positive episodes, however from existing reports, we propose a few theories: (1) the virus may have an intermittent dormancy period where the immune response and corresponding clinical symptoms caused by the infection have reduced, however the virus may continue to persist in patients, leading to positive nucleic acid detection results. Studies have shown that the median duration of the loss of SARS-CoV-2 from patients was 20 days, where the shortest duration was 8 days and the longest was 37 days when the virus could be detected in the body of a deceased patient until the time of death (15). However, another study observed that a COVID-19 patient displayed pulmonary absorption one week after admission; however, the throat swab test still yielded positive results. The patient tested positive for the virus for 49 days, however, the test result was negative on the 51st day after infection (16). The duration of re-positive episodes in the patients tracked in our study ranged from one week to one month, which may be related to the continuous release of the virus from the lower respiratory tract. However, the dormancy period and clearance characteristics of SARS-CoV-2 in the body of patients are still not fully understood. (2) Insufficient course of drug treatment and incomplete viral clearance: re-positive episodes may be due to the suppression of viral replication due to drugs utilised during hospitalization and insufficient or lower viral load during detection resulting in false-negative results. However, the actual virus may not have been completely cleared from the body. After discharge from the hospital, a decrease or suspension of the use of drugs prescribed may lead to an increase in the proliferation of the virus, leading to a positive nucleic acid test result. (3) Error in sample collection: Most nucleic acids detected by RT-PCR assays are obtained from nasal or throat swab samples. The location from where the sample is collected may be inaccurate, and the virus might not be captured in the sample despite following the procedure. Factors such as sample transportation, commissioning, and detection kits used also influence the test results. A study involving 610 patients with SARS-CoV-2 infection reported a high proportion of false-negative RT-PCR test results (16, 17). In our study, the six patients not only had negative results twice in a row, but also showed no symptoms of viral infection or signs of respiratory infection at the time of their first discharge. At the second admission, there were no symptoms as their chest CT scans were normal, and laboratory test data of re-admitted patients showed no significant differences compared to data from their first diagnosis. Therefore, initial false-negative test results have to be accounted for when studying re-positive cases determined by RT-PCR.

It has been reported that the proportion of re-positive in adolescents under 14 years of age is higher than the proportion over the age of 14 years; patients with severe symptoms during hospitalization were found to not develop re-positive episodes (17). However, in our follow-up case, there were 2 re-positive patients with severe symptoms, and our study found that there were no significant differences in age between re-positive patients and recovered patients, which may indicate that the likelihood of developing re-positive episodes is not closely related to the severity of the condition at the first diagnosis or age of the patients. In addition, studies have found that male patients generally have higher infection and death rates than female patients (18), which is believed to be due to the influence of hormones, and the higher expression of the viral receptor, angiotensin-converting enzyme 2 (ACE2) protein in males (19). In addition, studies have reported that non-coding RNA has an important regulatory role in cardiovascular disease (20, 21). As a cardiovascular regulator, ACE2 may play a potential role in the re-positive of COVID-19 and may be regulated by non-coding RNA, which is worthy of further development research. Our study found that there was no significant difference in the number of male and female re-positive patients, which may be because ACE2 does not play a key role in developing re-positive episodes. It is worth noting that due to the small number of cases selected in this study, and the concentration of cases in the Jiaodong Peninsula region, this study may only represent the characteristics of the re-positive cases in this region.

We found that serum creatinine level is closely related to whether patients will be re-positive or not. As a product of muscle metabolism, serum creatinine is metabolized out of the body through the kidney. Studies have found that the kidney’s ability to clear creatinine decreases with aging (22). Even though there were no significant differences in age and gender distribution between the re-positive and non-repositive groups, we found that male over the age of 45 appeared to account for a greater proportion of the re-positive patients. Age-related changes in creatinine levels, even if not out of the normal range, seem to have the potential to be a sensitive early predictor of the likelihood of re-positive. However, how does the change of aging and creatinine affect re-positive, which still needs more and more in-depth research to confirm and verify.

Eosinophils have powerful immune functions and are thought to be associated with disorders such as asthma and allergies. Studies have shown that severe respiratory syncytial virus infection is closely related to the recruitment and degranulation of eosinophils into the pulmonary parenchyma (23, 24), playing a key role in promoting tissue damage and bronchospasm. However, eosinophils are vital for immune function, especially for developing immunity against viral infections. Studies have confirmed that eosinophils have antiviral activity against respiratory syncytial virus *in vitro*, and it has been observed that eosinophil counts in the lungs are always elevated before the onset of disease, regardless of the respiratory virus initially inoculated (25). Studies have found that the percentage of eosinophils in patients diagnosed with COVID-19 is abnormally low, and by analysing the correlation of eosinophils, fever, and pneumonia, it was found that COVID-19 and fever are negatively correlated with the percentage of eosinophils, and the ratio of eosinophils to neutrophils (26). In addition, it has been reported that the duration of SARS-CoV-2 load is 3-14 days and eosinophil counts continue to 7-9 days, and eosinophilia may be an indicator of COVID-19 improvement (27). Existing studies have shown that eosinophils are associated with poor prognosis, and there is evidence that eosinophils are associated with acute respiratory deterioration (28–30). Consistently, the ICU metastasis rate was significantly higher in the eosinopenia group than in the non-eosinopenia group (31). Moreover, eosinophil reduction has been reported in patients with SARS-CoV-2 infection, which can be improved when the viral load in patients is reduced (27, 32), and a study have shown that the recovery of eosinophils has independent prognostic value for the course of mild diseases (33). One study tested the hypothesis that the decrease in eosinophils was associated with a malfunction of the innate immune response, and found that the decrease in eosinophils was specific for COVID-19 (34). In addition, a prospective observational study in France suggested that eosinopenia could be used as an adjuvant index for patients with suspected COVID-19, since it is more sensitive and specific than lymphocytes (35), and another study reached the same conclusion (36). In our study, we found that patients in the stages of initial infection and re-positive episodes display significantly decreased eosinophil counts. Consistent with previous research, this finding suggests that eosinophil counts are a reliable indicator of re-positive episodes, not only for the diagnosis and progression of COVID-19, but also for the continuous monitoring of patients after discharge.

In addition, this study had several limitations, and the biggest one is that sample size is too little, because there are only 7 re-positive cases from a relatively limited area are included. Secondly, because our research is retrospective, we have not been able to detect the information of the virus sequence. As a result, the comprehensive and in-depth research is necessary. Further research needs to include as many re-positive patients as possible in a wider area and need to detect virus sequence accordingly, so that the diagnostic value of eosinophils for re-positive patients can be more reliably confirmed, and it can also help clinicians to analyse and grasp more comprehensively characteristics of re-positive patients.

## Conclusion

In this study, it was found that the age, sex, and severity of the disease of patients at the time of initial diagnosis were not closely related to recurrence of COVID-19, however, male older than 45 years with comorbidities account for a greater proportion of the patients. There were significant differences in the creatinine levels of re-positive patients and recovered patients at the time of initial diagnosis. Although the patients showed clinical symptoms that were milder in the re-positive phase than in the initial diagnosis phase, clinical laboratory test results showed no significant difference in inflammatory indicators between the two phases. Regarding the clinical characteristics of re-positive patients, this study found that eosinophil counts decreased significantly in both the initial diagnosis and re-positive phases, whereas it increased normally in the negative phase. Similarly, the platelet counts of re-positive patients were significantly lower during the initial diagnosis and re-positive phases than during the negative phase.

In summary, the levels of creatinine and albumin may be able to help differentiate between a re-positive population at initial diagnosis, and eosinophils counts may play a role in determining the stage of the patient’s disease, but their role in viral immunity and re-positive patients needs further study. So these evidences give us a thought, does the proliferation of SARS-COV-2 virus cause eosinophilia reduction in the body? Is drug regulation of eosinophils potentially effective in controlling virus replication which could prevent for reinfection with SARS-COV-2 virus? Hopefully, these problems could attract more attention, and more studies are definitely needed for further investigation.

## Data Availability Statement

The raw data supporting the conclusions of this article will be made available by the authors, without undue reservation.

## Ethics Statement

Written informed consent was obtained from the individual(s), and minor(s)’ legal guardian/next of kin, for the publication of any potentially identifiable images or data included in this article.

## Author Contributions

All authors had full access to all the data in the study and take responsibility for the integrity of the data and the accuracy of the data analysis. TY, SJ, DY, and XL were responsible for study concept and design. YY, XL, XF, FJ, CB, and GM were responsible for the acquisition, analysis, or interpretation of data. ZB was responsible for collecting clinical data and revising the manuscript. XL and TY were responsible for drafting the manuscript. XL and TY were responsible for statistical analysis. All authors contributed to the article and approved the submitted version.

## Funding

This work was supported by the National Natural Science Foundation of China (grant no. 81870331) and the Qingdao Municipal Science and Technology Bureau Project (grant no. 21-1-4-rkjk-12-nsh).

## Conflict of Interest

The authors declare that the research was conducted in the absence of any commercial or financial relationships that could be construed as a potential conflict of interest.

## Publisher’s Note

All claims expressed in this article are solely those of the authors and do not necessarily represent those of their affiliated organizations, or those of the publisher, the editors and the reviewers. Any product that may be evaluated in this article, or claim that may be made by its manufacturer, is not guaranteed or endorsed by the publisher.

## References

[1] JNm. The Species Severe Acute Respiratory Syndrome-Related Coronavirus: Classifying 2019-Ncov and Naming it SARS-CoV-2. Nat Microbiol (2020) 5(4):536–44. 10.1038/s41564-020-0695-z PMC7095448 32123347

[2] COVID-19 Dashboard by the Center for Systems Science and Engineering (CSSE) at Johns Hopkins University (JHU). Available at: https://coronavirus.jhu.edu/map.html (Accessed June 7, 2020).

[3] Scudellari MJN. How the Pandemic Might Play Out in 2021 and Beyond. Nature (2020) 584(7819):22–5. 10.1038/d41586-020-02278-5 32760050

[4] Notice on the Issuance of the COVID-19 Protocol (Trial Seventh Edition). Available at: http://www.nhc.gov.cn/yzygj/s7653p/202003/46c9294a7dfe4cef80dc7f5912eb1989.shtml (Accessed March 3, 2020).

[5] Lan L Xu D Ye G Xia C Wang S Li Y. Positive RT-PCR Test Results in Patients Recovered From COVID-19. JAMA (2020) 323(15):1502–3. 10.1001/jama.2020.2783 PMC7047852 32105304

[6] Peng J Wang M Zhang G Lu E. Seven Discharged Patients Turning Positive Again for SARS-CoV-2 on Quantitative RT-PCR. Am J Infect Control (2020) 48(6):725–6. 10.1016/j.ajic.2020.03.017 PMC7151314 32317126

[7] Zhang B Liu S Dong Y Zhang L Zhong Q Zou Y. Positive Rectal Swabs in Young Patients Recovered From Coronavirus Disease 2019 (COVID-19). J Infect (2020) 81(2):e49–e52. 10.1016/j.jinf.2020.04.023 32335176 PMC7177113

[8] Chen D Xu W Lei Z Huang Z Liu J Gao Z. Recurrence of Positive SARS-CoV-2 RNA in COVID-19: A Case Report. Int J Infect Dis (2020) 93:297–9. 10.1016/j.ijid.2020.03.003 PMC7129213 32147538

[9] Li Y Hu Y Yu Y Zhang X Li B Wu J. Positive Result of Sars-Cov-2 in Faeces and Sputum From Discharged Patients With COVID-19 in Yiwu, China. J Med Virol (2020) 92(10):1938–47. 10.1002/jmv.25905 PMC7264799 32311109

[10] Bhargava A Fukushima E Levine M Zhao W Tanveer F Szpunar S. Predictors for Severe COVID-19 Infection. Clin Infect Dis (2020) 71(8):1962–8. 10.1093/cid/ciaa674 PMC7314166 32472676

[11] Chan A Rout A. Use of Neutrophil-To-Lymphocyte and Platelet-To-Lymphocyte Ratios in COVID-19. J Clin Med Res (2020) 12(7):448–53. 10.14740/jocmr4240 PMC7331861 32655740

[12] Wang M Zhu Q Fu J Liu L Xiao M Du. Differences of Inflammatory and non-Inflammatory Indicators in Coronavirus Disease-19 (COVID-19) With Different Severity. Infect Genet Evol (2020) 85:104511. 10.1016/j.meegid.2020.104511 32858231 PMC7448737

[13] Zangrillo A Landoni G Beretta L Morselli F Serpa Neto A Bellomo R. Angiotensin II Infusion in COVID-19-Associated Vasodilatory Shock: A Case Series. Crit Care (2020) 24(1):227. 10.1186/s13054-020-02928-0 32414393 PMC7228670

[14] Mulligan MJJ. An Inactivated Virus Candidate Vaccine to Prevent COVID-19. JAMA (2020) 324(10):943–45. 10.1001/jama.2020.15539 32789500

[15] Zhang L Sun W Wang Y Wang X Liu Y Zhao S. Clinical Course and Mortality of Stroke Patients With Coronavirus Disease 2019 in Wuhan, China. Stroke (2020) 51(9):2674–82. 10.1161/STROKEAHA.120.030642 PMC7434009 32755348

[16] Tan L Kang X Zhang B Zheng S Liu B Yu T. A Special Case of COVID-19 With Long Duration of Viral Shedding for 49 Days. medRxiv (2020):2020.03.22.20040071. 10.1101/2020.03.22.20040071 PMC7262084 34172984

[17] An J Liao X Xiao T Qian S Yuan J Ye H. Clinical Characteristics of the Recovered COVID-19 Patients With Re-Detectable Positive RNA Test. Ann Transl Med (2020) 8(17):1084. 10.1101/2020.03.26.20044222 33145303 PMC7575971

[18] Jin J Bai P He W Wu F Liu X Han D. Gender Differences in Patients With COVID-19: Focus on Severity and Mortality. Front Public Health (2020) 8:152. 10.3389/fpubh.2020.00152 32411652 PMC7201103

[19] Ghazizadeh Z Majd H Richter M Samuel R Zekavat S Asgharian H. Androgen Regulates SARS-CoV-2 Receptor Levels and Is Associated With Severe COVID-19 Symptoms in Men. Cell Stem Cell (2020) 27(6):876–89.e12. 10.1101/2020.05.12.091082 33232663 PMC7670929

[20] He X Yang Y Wang Q Wang J Li S Li C. Expression Profiles and Potential Roles of Transfer RNA-Derived Small RNAs in Atherosclerosis. J Cell Mol Med (2021) 25(14):7052–65. 10.1111/jcmm.16719 PMC8278088 34137159

[21] Fu X He X Yang Y Jiang S Wang S Peng X. Identification of Transfer RNA-Derived Fragments and Their Potential Roles in Aortic Dissection. Genomics (2021) 113(5):3039–49. 10.1016/j.ygeno.2021.06.039 34214628

[22] Lim J Kim E Kim M Chung S Shin S Kim H. Age-Associated Molecular Changes in the Kidney in Aged Mice. Oxid Med Cell Longev (2012) 2012:171383. 10.1155/2012/171383 23326623 PMC3544311

[23] Harrison A Bonville C Rosenberg H Domachowske J. Respiratory Syncytical Virus-Induced Chemokine Expression in the Lower Airways: Eosinophil Recruitment and Degranulation. Am J Respir Crit Care Med (1999) 159(6):1918–24. 10.1164/ajrccm.159.6.9805083 10351940

[24] Garofalo R Kimpen J Welliver R Ogra P. Eosinophil Degranulation in the Respiratory Tract During Naturally Acquired Respiratory Syncytial Virus Infection. J Pediatr (1992) 120(1):28–32. 10.1016/S0022-3476(05)80592-X 1731020

[25] Rosenberg H Domachowske J. Eosinophils, Eosinophil Ribonucleases, and Their Role in Host Defense Against Respiratory Virus Pathogens. J Leukoc Biol (2001) 70(5):691–8. 11698487

[26] Yang J Zhao X Liu X Sun W Zhou L Wang Y. Clinical Characteristics and Eosinophils in Young SARS-CoV-2-Positive Chinese Travelers Returning to Shanghai. Front Public Health (2020) 8:368. 10.3389/fpubh.2020.00368 32754569 PMC7365885

[27] Liu F Xu A Zhang Y Xuan W Yan T Pan K. Patients of COVID-19 may Benefit From Sustained Lopinavir-Combined Regimen and the Increase of Eosinophil may Predict the Outcome of COVID-19 Progression. Int J Infect Dis (2020) 95:183–91. 10.1016/j.ijid.2020.03.013 PMC7193136 32173576

[28] Lindsley A Schwartz J Rothenberg M. Immunology C. Eosinophil Responses During COVID-19 Infections and Coronavirus Vaccination. J Allergy Clin Immunol (2020) 146(1):1–7. 10.1016/j.jaci.2020.04.021 32344056 PMC7194727

[29] Georgakopoulou V Garmpis N Damaskos C Valsami S Dimitroulis D Diamantis E. The Impact of Peripheral Eosinophil Counts and Eosinophil to Lymphocyte Ratio (ELR) in the Clinical Course of COVID-19 Patients: A Retrospective Study. In Vivo (2021) 35(1):641–8. 10.21873/invivo.12303 PMC7880766 33402521

[30] Xie G Ding F Han L Yin D Lu H Zhang MJA. The Role of Peripheral Blood Eosinophil Counts in COVID-19 Patients. Allergy (2021) 76(2):471–82. 10.1111/all.14465 PMC7323233 32562554

[31] Huang J Zhang Z Liu S Gong C Chen L Ai G. Absolute Eosinophil Count Predicts Intensive Care Unit Transfer Among Elderly COVID-19 Patients From General Isolation Wards. Front Med (Lausanne) (2020) 7:585222. 10.3389/fmed.2020.585222 33251234 PMC7673383

[32] Mu T Yi Z Wang M Wang J Zhang C Chen H. Expression of Eosinophil in Peripheral Blood of Patients With COVID-19 and its Clinical Significance. J Clin Lab Anal (2021) 35(1):e23620. 10.1002/jcla.23620 33118666 PMC7645967

[33] Mateos González M Sierra Gonzalo E Casado Lopez I Arnalich Fernández F Beato Pérez J Monge Monge D. The Prognostic Value of Eosinophil Recovery in COVID-19: A Multicentre, Retrospective Cohort Study on Patients Hospitalised in Spanish Hospitals. J Clin Med (2021) 10(2):305. 10.3390/jcm10020305 33467585 PMC7830154

[34] Koenderman L Siemers M van Aalst C Bongers S Spijkerman R Bindels B. The Systemic Immune Response in COVID-19 Is Associated With a Shift to Formyl-Peptide Unresponsive Eosinophils. Cells (2021) 10(5):134952. 10.20944/preprints202102.0453.v1 PMC8147959 34062964

[35] Outh R Boutin C Gueudet P Suzuki M Saada M Aumaître HJ. Eosinopenia <100/μl as a Marker of Active COVID-19: An Observational Prospective Study. J Microbiol Immunol Infect (2021) 54(1):61–8. 10.1016/j.jmii.2020.12.005 PMC7792500 33468435

[36] Tan Y Zhou J Zhou Q Hu L Long YJ. Role of Eosinophils in the Diagnosis and Prognostic Evaluation of COVID-19. J Med Virol (2021) 93(2):1105–10. 10.1002/jmv.26506 32915476

